# Endoplasmic reticulum stress participates in the pathophysiology of mercury-caused acute kidney injury

**DOI:** 10.1080/0886022X.2019.1686019

**Published:** 2019-11-18

**Authors:** Plácido Rojas-Franco, Margarita Franco-Colín, Alejandra Paola Torres-Manzo, Vanessa Blas-Valdivia, María del Rocio Thompson-Bonilla, Sinan Kandir, Edgar Cano-Europa

**Affiliations:** a Laboratorio de Metabolismo I Departamento de Fisiología, Escuela Nacional de Ciencias Biológicas, Instituto Politécnico Nacional, Ciudad de México, México;; b Department of Molecular Physiology and Biophysics, Vanderbilt University, Nashville, TN, USA;; c Laboratorio de Neurobiología, Departamento de Fisiología, Escuela Nacional de Ciencias Biológicas, Instituto Politécnico Nacional, Ciudad de México, México;; d Laboratorio de Medicina Genómica. Hospital Regional 1ro. de Octubre. ISSSTE, Ciudad de México, México;; e Department of Physiology, Ceyhan Faculty of Veterinary Medicine, Cukurova University, Adana, Turkey

**Keywords:** Mercury nephrotoxicity, acute kidney injury, mercury chloride, endoplasmic reticulum stress, unfolded protein response

## Abstract

Acute exposure to mercury chloride (HgCl_2_) causes acute kidney injury (AKI). Some metals interfere with protein folding, leading to endoplasmic reticulum stress (ERS), and the activation of cell death mechanisms, but in the case of mercury, there is no knowledge about whether the ERS mediates tubular damage. This study aimed to determinate if HgCl_2_ causes an AKI course with temporary activation of ERS and if this mechanism is involved in kidney cell death. Male mice were intoxicated with 5 mg/kg HgCl_2_ and sacrificed after 24, 48, 72, and 96 h of mercury administration. The kidneys of euthanized mice were used to assess the renal function, oxidative stress, redox environment, antioxidant enzymatic system, cell death, and reticulum stress markers (PERK, ATF-6, and IRE1α pathways). The results indicate temporary-dependent renal dysfunction, oxidative stress, and an increase of glutathione-dependent enzymes involved in the bioaccumulation process of mercury, as well as the enhancement of caspase 3 activity along with IRE1a, GADD-153, and caspase 12 expressions. Mercury activates the PERK/eIF2α branch during the first 48 h. Meanwhile, the activation of PERK/ATF-4 branch allowed for ATF-4, ATF-6, and IRE1α pathways to enhance GADD-153. It led to the activation of caspases 12 and 3, which mediated the deaths of the tubular and glomerular cells. This study revealed temporary-dependent ERS present during AKI caused by HgCl_2_, as well as how it plays a pivotal role in kidney cell damage.

## 1. Introduction

Mercury (Hg) is a heavy metal used in the agriculture, mining, pharmaceutical, and chemistry industries. It exists in many chemical forms as organic salts (methyl- or ethyl-mercury), inorganic salts (HgCl_2_), or a vapor [1,2]. Over time, several intoxications with all chemical forms of this metal have been documented. However, inorganic mercury salts produce greater renal damage than other mercury chemical forms do. Particularly, HgCl_2_ causes acute kidney injury (AKI), and it is the most common model for studying renal toxicity mechanisms [3–5].

Several biochemical mechanisms are involved in cell injury caused by mercury. One of the most crucial mercury toxicity mechanisms is its binding to the sulfhydryl group present in proteins. Also, HgCl_2_ reduced glutathione (GSH), cysteine (Cys), and homocysteine (Hcy) concentration into the cells. In the kidney, some transport mechanisms bioaccumulate mercury complexes with thiols, such as organic anion transporters 1 and 3 (OAT1/OAT3) and system b^0,+^. It causes oxidative stress, cytoskeletal structure alterations, and cell damage [6,7]. Also, HgCl_2_ enhances the Sirt1/Nrf2/OH-1 pathway, which does not compensate for oxidative stress. Moreover, mercury chloride reduces ATP synthesis and the activities of Na^+^-K^+^-ATPase, Mg^2+^-ATPase, Ca^2+^-ATPase, and Ca^2+^-Mg^2+^-ATPase. If the sodium and calcium gradients change, the kidney cells suffer cell death characterized by tubular necrosis, glomerular sclerosis, and interstitial inflammatory cell infiltration [5,8].

Also, some reports link mercury poisoning with organelle dysfunction, such as mitochondria, cytoskeleton, and endoplasmic reticulum, both *in vivo* and *in vitro* [7–10]. However, concerning the endoplasmic reticulum, a pathway that correlates this organelle with AKI caused by mercury has not been described. Still, some reports show that mercury and other heavy metals can interfere with proper protein folding in the endoplasmic reticulum, which can be a significant detriment to cell survival [11]. Unfolded or misfolded protein accumulation is associated with several cellular stressors, such as redox environment disturbance, a Ca^2+^ imbalance, altered protein glycosylation, or protein folding defects; it is known as endoplasmic reticulum stress (ERS) [12]. Meanwhile, the unfolded protein response (UPR) is a process that seeks to restore the endoplasmic reticulum’s normal function through multiple strategies mediated by the initial activation of ER membrane-associated sensors, PKR-like ER-kinase (PERK), activating transcription factor 6 (ATF6), and the inositol-requiring enzyme-1alpha (IRE1α). When the UPR is not enough to restore the organelle homeostasis, the cells activate cell-death mechanisms that are primarily mediated by the action of the UPR protein growth arrest, the deoxyribonucleic acid damage-inducible gene 153 (GADD-153, also known as CHOP), and ER membrane-associated caspase 12 [12,13].

Nowadays, there are no reports about the relationship between AKI and ERS caused by mercury chloride. However, we propose that there is a direct association between mercury and ERS in the kidney because in an *in vitro* model using an NRK-52E kidney cell culture, HgCl_2_ increases the GRP78 expression (an ERS sensor) [14]. Also, in the brain, there are studies to correlate the three pathways of ERS (IRE1α, ATF-4, PERK) and organic mercury toxicity [15].

Thus, the aim of this study was the establishment of a temporal relationship between AKI caused by HgCl_2_ and the processes of oxidative stress, ERS, and cell death.

## 2. Materials and methods

### 2.1. Animals housing conditions and experimental design

We used 60 albino male mice between 25 and 30 g. They housed in a cooled room (21 ± 2 °C) with 12/12-h light cycles, relative humidity of 40–60%, and food and water *ad libitum*. All experimental procedures described in this study follow the Mexican Official Standard NOM-082-ZOO-1999 as well as the Guide for the Care and Use of Laboratory Animals from the National Research Council (US) Committee (National Research Council (US) Committee for the Update of the Guide for the Care and Use of Laboratory Animals, 2011). Also, the protocol received approval from the Internal Bioethics Committee (CEI-ENCB) with number approbation CEI-ENCB 019/2014.

The animals were divided into two groups: the control group (*n* = 12) received a 0.9% saline solution (SS) intraperitoneally (ip), and the treated group (*n* = 48) received a single dose of HgCl_2_ (5 mg/kg; ip). Twelve animals from each group were euthanized with pentobarbital monosodic (90 mg/kg ip) at 24, 48, 72, and 96 h after mercury intoxication. Meanwhile, the control group was euthanized at 96 h using the same drug. Before euthanizing, six animals underwent renal function evaluation using a metabolic cage for 6 h without food and water as previously described [16]. The urine and serum samples were used to evaluate renal function. The next six animals from each group were euthanized without renal function evaluation, and we dissected the kidneys. The left kidneys were frozen and kept at −70 °C until use, whereas the right kidneys were fixed in 4% paraformaldehyde in PBS.

### 2.2. Renal function evaluation

We employed a commercially available assay kit (*Spinreact* company, Girona, Spain) to evaluate the glucose, creatinine, and protein in the urine, as well as the BUN, uric acid, and creatinine in serum.

### 2.3. Biochemical and molecular determinations

We used frozen kidneys homogenized in 3 mL of 10 mM of phosphate buffer pH 7.4, and then, they were used to assess all of the oxidative stress markers, the enzymatic activities, the caspase 3 activity, and the western blot assays. The protein concentration was determined by using the Bradford method [17].

### 2.4. Quantification of oxidative stress markers

We assessed the lipid peroxidation (LP), reactive oxygen species (ROS), oxidized glutathione (GSSG), and nitrite (NO_2_) quantifications as oxidative stress markers as previously described with some modification [18,19].

### 2.5. Evaluation of the antioxidant enzymatic system (SOD, catalase, and total SOD) and some enzymes associated with the toxicity of mercury (glutathione-S-transferase, γ-glutamyl transpeptidase and myeloperoxidase)

Spectrophotometrical techniques were used to evaluate all enzyme activities as previously described [18,20,21] with some modification to the microplate evaluation with the Multiskan GO (Thermo Scientific, Waltham, MA).

For glutathione reductase (GR) activity, 2 μL of the homogenate was added to 100 μL to 100 mM of phosphate buffer (pH 7.0) containing 1 mM of GSSG and 0.1 mM of NADPH. The reaction was monitored at a temperature of 37 °C for 10 min. The results are expressed as mmoles of NADPH used/mg protein/min.

For the catalase activity, we used 5 μL of cell extract to 3 mL of 100 mM phosphate buffer, pH 7.4, containing 30 mM of H_2_O_2_. The absorbance was recorded at 240 nm after 10 min at 37 °C. The decomposition of H_2_O_2_ by the catalase present in the samples followed first-order kinetics according to the equation of *k* = 2.3 *t* log *A*o/*A*, where *k* is the first-order reaction rate constant, *t* is the time over which the decrease of hydrogen peroxide caused by catalase activity was measured (10 min), and *A*o/*A* is the ratio of optical densities at times 0 and 10 min. Catalase activity is expressed as k/mg of protein.

The SOD activity was measured spectrophotometrically; 5 μL of cell extract was mixed with 2.9 mL of a solution containing 10 μm NaN_3_, 10 μm of reduced cytochrome c, and 1 mM of EDTA dissolved in 20 mM of sodium bicarbonate and 0.02% Triton X-100, pH 10.2. The enzymatic assay was started by the addition of 50 μL of xanthine oxidase (3.4 mg/mL in 0.1 mM of EDTA). The change in absorbance was monitored every 30 s at 550 nm for 3 min. One unit of SOD activity is the amount of enzyme that decreased the reduction rate of cytochrome c by 50%. The SOD activity is expressed as U/mg of protein for glutathione-S-transferase (GST). 5 μL of the homogenate was added to 100 μL of 0.1 M of Tris–HCl, pH 8.2, containing 10 mM of GSH. The mixture was incubated for 1 min at 37 °C. After that, 5 μL of 50 mM of 1-chloro-2,4-dinitrobenzene was added, and we monitored the absorbance at 340 nm for 3 min. One unit (U) of GST caused the formation of 1 μmol of S-2,4-dinitrophenyl glutathione, using its molar extinction coefficient (*ε*_340_ = 10 mM^−1^cm^−1^). Thus, GST activity is expressed as U/mg protein/min.

For γ-glutamyl transpeptidase (γ-GT), we used 5 μL of the cell homogenate, which was added to 100 μL of 100 mM of Tris–HCl, pH 8.0, containing 1 mM of γ-glutamyl *p*-nitroaniline and 20 mM of glycylglycine. The mixture was incubated at 37 °C, and we monitored the absorbance at 410 nm for 4 min. The molar extinction coefficient of p-nitroaniline formed was used to express the results as mmol p-nitroaniline formed/mg protein/min (ε_410_ = 8800M^−1^cm^−1^).

For myeloperoxidase (MPO) activity, 2 μL of the homogenate was added to 160 μL of 90 mM of a citrate buffer (pH 4.5) containing 0.1% Triton X-100 and 0.65 mM of O-dianisidine. The reaction started with the addition of 40 μL of 0.43 mM of H_2_O_2_. The mixture was incubated at 37 °C. We monitored the absorbance at 460 nm for 15 min, and at 460 nm for 5 min. The molar extinction coefficient of p-nitroaniline formed was used to express the results as mmoles O-dianisidine oxidized/mg protein/min (ε_460_ = 11.3 mM^−1^cm^−1^).

### 2.6. Endoplasmic reticulum stress evaluation

We evaluated the ERS by quantifying the expression of some intermediaries from the PERK, ATF-6, and IRE1 pathways through western blot assays as previously described [18]. Briefly, 50 μg of protein was charged in 10% polyacrylamide gels and separated by electrophoresis, then electrotransferred to PVDF membranes in a semidry chamber. After that, proteins of gels were electrotransferred to PVDF membranes in a semidry chamber. Transferred membranes were blocked for 1 h under constant stirring in PBST (PBS with 0.05% tween 20 with 5% low-fat milk). Blocked membranes were incubated at 4 °C overnight in a blocking buffer containing the primary antibodies of ATF-6α, GADD34, XBP-1, GADD 153, eIF2α (Santa Cruz Biotechnology, Santa Cruz, CA: sc-22799, sc-8327, sc-575 and sc-7160, sc-30882, respectively), and caspase-12 (Millipore, AB3613, Burlington, MA) diluted 1:500, as well as IRE1α Abcam, ab37073) and ATF-4 (Biorbyt, orb-129518) diluted 1:1000 and 1:2000, respectively. After incubation, blots were washed three times with fresh PBST (20 min. per wash) and then incubated in a 1:2000 diluted secondary antibody (HPR-conjugated goat anti-rabbit or rabbit anti-goat; Life technologies, Rockford, IL; 65-6120, 611620, respectively) for 1 h at room temperature under constant stirring. Then, membranes were washed three times with PBST, and finally, protein bands were revealed in photographic plates (JUAMA, México) by chemiluminescence, using Luminata TM Forte from Millipore). Protein β-actin expression was used as a charge control and constitutive protein (Santa Cruz Biotechnology; sc-1615, dilution: 1:4000). The optical density (O. D.) from all bands obtained was analyzed by Image J program (NIH, Bethesda, MD; version 1.51p), according to program specifications. Proteins O. D. are expressed as a protein/β-actin ratio.

### 2.7. Caspase 3 activity assay

Caspase 3 activity was assessed by using a commercial caspase 3 colorimetric assay kit (Millipore, APT165) as previously described [16].

### 2.8. Histological analysis

The kidney fixed with paraformaldehyde was embedded in paraffin, and 5-μm slices were obtained with a standard microtome. One section was stained with hematoxylin–eosin (HE), dehydrated, and mounted in resin. Meanwhile, another section was incubated with annexin V-Cy3 (100 µg/ml, Millipore) for 15 min in darkness. The photomicrographs were acquired with Nikon-50i, and they were digitalized and analyzed with an NIS element program.

### 2.9. Statistical analysis

All data are presented as mean ± standard error of the mean, and they were analyzed by one-way analysis of variance (one-way ANOVA) and the Student–Newman–Keuls *post hoc* test. Values with *p* < 0.05 were considered to be statistically different.

## 3. Results

Table 1 shows the effect of HgCl_2_ on renal function markers during the progress of HgCl_2_ intoxication. It shows an increase of BUN, serum uric acid, serum creatinine, glycosuria, and proteinuria with a reduction in the clearance creatinine since 24 h after mercury intoxication.

**Table 1.** Temporal evaluation of functional renal markers in the acute kidney injury induced with HgCl_2_.

Group	Serum BUN (mg/dL)	Serum uric acid (mg/dL)	Urine glucose (mg/dL)	Urine proteins (mg/dL)	Serum creatinine (mg/dL)	Urine cretinine (mg/dL)	Creatinine clearence (mL/min)
HgCl_2_							
Control	31.94 ± 4.28^a^	2.08 ± 0.14^a^	55.03 ± 6.48^a^	7.46 ± 0.70^a^	1.24 ± 0.19^a^	28.35 ± 8.22	0.75 ± 0.23^a^
24 h	73.10 ± 10.9^b^	4.53 ± 0.08^b^	113.7 ± 4.43^b^	22.28 ± 4.63^b^	2.58 ± 0.45^b^	48.89 ± 7.13	0.17 ± 0.068^b^
48 h	81.14 ± 4.13^b,c^	5.44 ± 0.61^b,c^	423 ± 74.94^c^	21.94 ± 2.82^b^	2.41 ± 0.31^b^	60.56 ± 8.40	0.24 ± 0.036^b^
72 h	123 ± 11.32^d^	5.65 ± 0.21^b,d^	308.5 ± 100.8^c,d^	19.46 ± 2.11^b^	2.06 ± 0.12^b^	30 ± 10.75	0.26 ± 0.029^b^
96 h	190 ± 27.14^e^	5.46 ± 0.19^b,e^	451.2 ± 8.63^c,e^	17.57 ± 0.87^b^	3.56 ± 0.71^c^	30.67 ± 14.66	0.13 ± 0.034^b^
*p*	<0.001	<0.001	0.012	0.003	0.012	0.128	0.004
*F*_4,25_	17.733	22.759	4.039	5.217	4.039	1.983	5.125

Data represent the mean ± SEM. a ≠ b ≠ c ≠ d ≠ e. One-way ANOVA, Student–Newman–Keuls *post hoc* (*n* = 6).

Table 2 shows the quantification of ROS, lipid peroxidation (LP) and nitrites (NO_2_), and glutathione oxidized (GSSG) in the kidney. The GSSG was the first metabolite that increased by about 37–56% from 24 h to 96 h after HgCl_2_ intoxication. The ROS and NO_2_ levels enhanced from 48 h after mercury administration about threefold and twofold, respectively. Meanwhile, HgCl_2_ caused an increase of the LP at 72 and 96 h, about 150 and 212%, respectively.

**Table 2.** Temporal evaluation of the oxidative stress markers in the acute kidney injury induced with HgCl_2_.

Group	Lipid peroxidation (URF/mg protein)	ROS quantification (ng of DCF/mg protein/h)	GSSG content (µg of GSSG/mg protein	NO_2_ quantification µg ofNO_2_/mg protein
Control	1.023 ± 0.003^a^	338.50 ± 32.18^a^	1.63 ± 0.09^a^	0.312 ± 0.01^a^
HgCl_2_				
24 h	1.146 ± 0.090^a^	435.51 ± 126.60^a^	2.24 ± 0.13^b^	0.404 ± 0.07ª
48 h	1.220 ± 0.040^a^	658.52 ± 5.78^b^	2.55 ± 0.20^b^	0.514 ± 0.03^b^
72 h	2.565 ± 0.230^b^	617.80 ± 2.41^b^	2.52 ± 0.14^b^	0.519 ± 0.02^b^
96 h	3.200 ± 0.100^c^	854.10 ± 18.59^c^	2.33 ± 0.13^b^	0.575 ± 0.06^b^
*p*	<0.001	<0.001	<0.001	0.002
*F*_4,25_	67.284	11.702	6.819	5.625

Data represent the mean ± SEM. a ≠ b ≠ c. One-way ANOVA, Student–Newman–Keuls *post hoc* (*n* = 6).

Table 3 shows the results of antioxidant enzyme activity. Catalase presented a time-dependent decay in its activity, whereas GR and MPO progressively raised their activities between 60 and 500%, respectively. We found a twofold increase in γGT activity starting at 24 h after intoxication. Meanwhile, GST presented a two-fold increased activity only at 48 h but remained unchanged for the rest of the time. The total SOD was not affected by HgCl_2_ intoxication during the 96 h after intoxication.

**Table 3.** Temporal evaluation of the antioxidant enzymatic system and the enzymes involved in the acute kidney injury induced with HgCl_2_.

Group	Glutatione reductase activity (mmol of NADPH consumed/mg protein/min)	Catalase activity (*k*/mg protein)	Total SOD (U SOD/mg protein)	GST activity (U GST/mg protein)	γ-GT activity (mmol of NBA formed/mg protein/min)	MPO activity (mmol of o-dianisidine oxidized/mg protein/min)
HgCl_2_						
Control	35.53 ± 2.21^a^	1.74 ± 0.04^a^	41.82 ± 4.99^a^	1.75 ± 0.09^a^	0.525 ± 0.06^a^	0.05 ± 0.001ª
24 h	42.76 ± 1.84^b^	0.93 ± 0.02^b^	38.52 ± 9.36ª	1.80 ± 0.28ª	1.064 ± 0.10^b^	0.13 ± 0.005^b^
48 h	46.10 ± 2.1^b^	0.92 ± 0.01^b^	44.13 ± 6.01ª	2.93 ± 0.36^b^	0.786 ± 0.01^b^	0.14 ± 0.003^b^
72 h	45.40 ± 2.95^b^	0.99 ± 0.03^b^	44.25 ± 1.90ª	2.48 ± 0.18ª	0.712 ± 0.02^b^	0.10 ± 0.003^c^
96 h	56.71 ± 2.30^c^	0.69 ± 0.01^c^	41.09 ± 10.50ª	1.72 ± 0.37ª	0.926 ± 0.14^b^	0.30 ± 0.012^d^
*p*	<0.001	<0.001	0.979	0.014	<0.001	<0.001
*F*_4,25_	10.908	258.27	0.107	3.839	6.274	234.840

Data represent the mean ± SEM. a ≠ b ≠ c. One-way ANOVA, Student–Newman–Keuls *post hoc* (*n* = 6).

**Figure 1.**
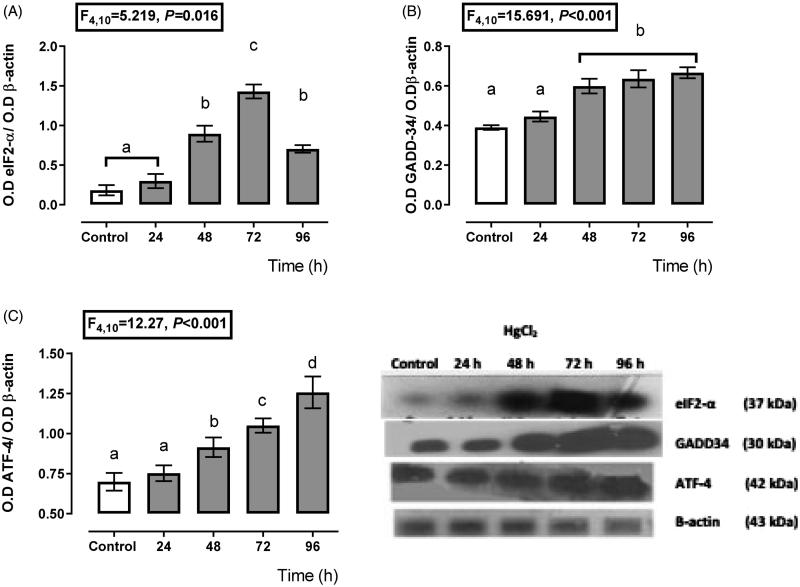
Time-dependent expression of endoplasmic reticulum stress markers, eIF2-α (A), GADD-34 (B), and ATF-4 (C) in the HgCl_2_-associated acute kidney injury. Data represents the mean ± SEM. One-way repeated measures ANOVA. Student–Newman–Keuls *post hoc* test. *n* = 6 a ≠ b ≠ c ≠ d ≠ e.

Figure 1 shows that the PERK branch had a high activity from 24 h to 72 h after mercury intoxication because we found a higher expression of eIF2α (Figure 1(A)) from 48 h (44% more expression than control) to 96 h (64% more expression than control) with a maximum expression at 72 h (110% compared with the control expression) after mercury intoxication. Also, the GADD-34 expression (Figure 1(B)) had higher expression with respect to the control from 24 h about 21%, with maximum expression at 72 h after mercury chloride intoxication about 89% with respect to the control group. Concerning ATF-4 (Figure 1(C)), its expression increased since 48 h after HgCl_2_ administration (30% more expression than the control group) until 96 h (79% more expression than control).

**Figure 2.**
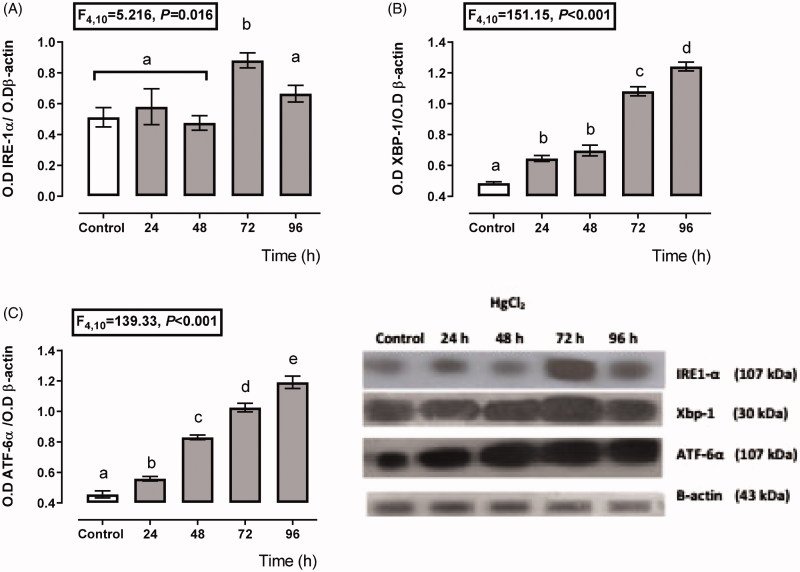
Time-dependent expression of endoplasmic reticulum stress markers IRE-1α (A), XBP-1 (B), and ATF-6α (C) in the HgCl_2_-associated acute kidney injury. Data represents the mean ± SEM. One-way ANOVA. Student–Newman–Keuls *post hoc* test. *n* = 6 a ≠ b ≠ c ≠ d ≠ e.

Figure 2 shows a transient depending over-expression of the IRE-1α and ATF6 branches. The expression of IRE-1α (Figure 2(A)), XBP-1 (Figure 2(B)), and ATF6 (Figure 2(C)) increased about 112–198%, 32–155%, and 22–161%, respectively, from 24 to 96 h after HgCl_2_ administration. However, the higher expression for XBP-1 and ATF6 was after 96 h of mercury intoxication (about 155 and 161% overexpression with respect to the control group); meanwhile, the higher IRE-1α expression was at 72 h (198%), and it decayed at 96 h (118%) after mercury intoxication.

**Figure 3.**
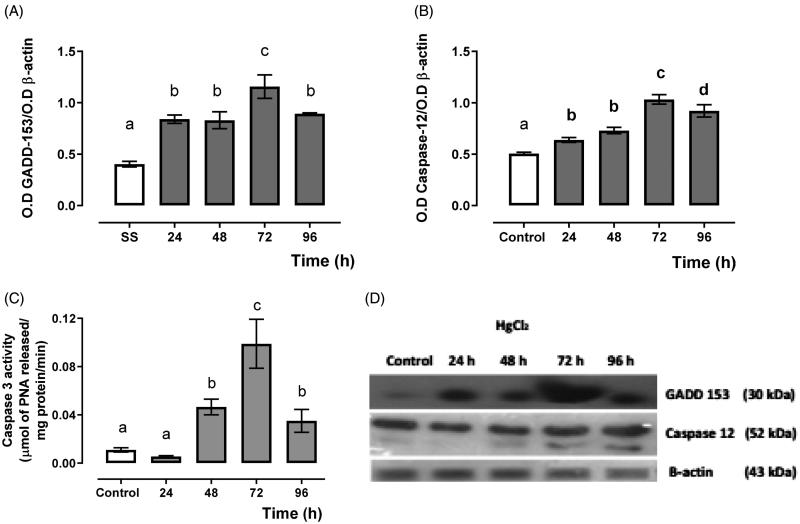
Time-dependent GADD-153 (A), caspase 12 expression (B) and caspase 3 activity (C), and representative blots of the Western blot technique, and (D) in the HgCl_2_-associated acute kidney injury. Data represents the mean ± SEM. One-way ANOVA. Student–Newman–Keuls *post hoc* test. *n* = 6 a ≠ b ≠ c ≠ d.

Figure 3 shows the expression of cell-death promoter protein GADD-153 (Figure 3(A)) and endoplasmic reticulum-associated caspase 12 (Figure 3(B)) and the effector caspase 3 activity (Figure 2(C)). It showed a higher expression for GADD-153 and caspase 12 as well as effector caspase 3 activity from 24 to 72 h, and they decayed at 96 h after mercury intoxication.

**Figure 4.**
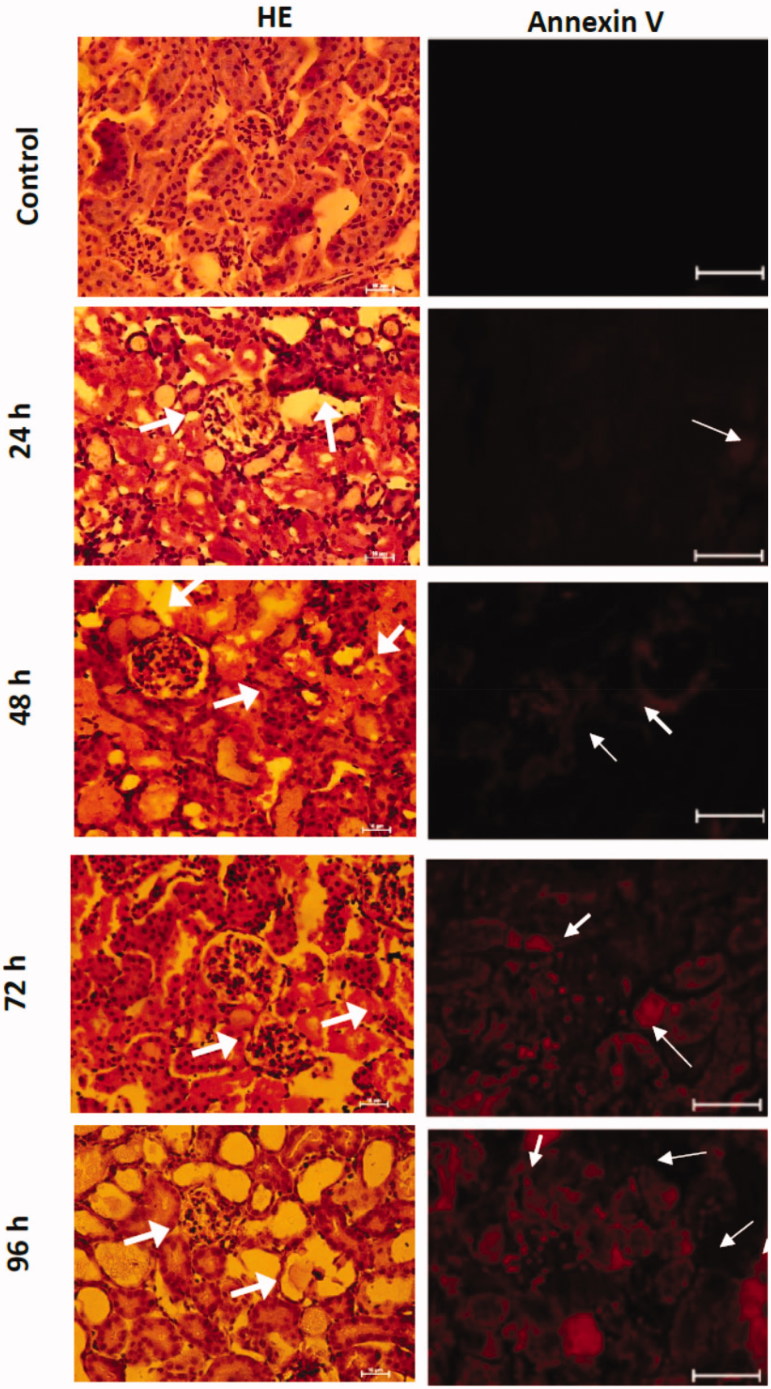
Temporal evaluation of the morphological changes in the HgCl_2_-associated acute kidney injury. The arrows show in HE stain photomicrographs the cellular atrophy of distal and proximal tubules, distortion of cellular continuity, nucleus loss, and hyperchromatic nuclei. Meanwhile, the annexin V positive cells are observed in red.

Also, Figure 4 shows the histological analysis of the kidney of the mice intoxicated with inorganic mercury. It showed transient cellular damage since 24 h after mercury intoxication. The damage observed in the hematoxylin–eosin stain was edema, cellular atrophy of distal and proximal tubules, distortion of cellular continuity, nucleus loss, and hyperchromatic nuclei. Even so, the immunofluorescence against annexin-V marked by a red fluorescent marker presented a transient overexpression for 24 h.

## 4. Discussion

Some reports demonstrate the relationship between mercury toxicity and ERS. It is well known that mercury can bind to natïve proteins and inhibit their biological activity by oxidizing and covalently bound with sulfhydryl groups from functional side chains, or also by displacing enzymes cofactors. All of these events promote renal dysfunction; our results show that HgCl_2_ causes AKI because it reduces in a temporary course the clearance creatine with an increase of uric acid, BUN, glycosuria, and proteinuria.

One of the responses of the kidney cells against mercury chloride intoxication is the overexpression of heat-shock proteins (HSP) and glucose-regulated proteins (GRP), with the purpose of enhancing the degradation of misfolded and unfolded proteins. Additionally, it increases the expression of genes encoding many other ER proteins to prevent the excessive accumulation of unfolded or misfolded proteins. The result above reveals that mercury enhanced the expression of these proteins as GADD34, and it is in accordance with other research groups that found that in the rat, the administration of HgCl_2_ enhances the expression of HSP72, HSP60, and GRP75, which are critical players in the assembling, transport, and refolding of proteins during oxidative damage [14,22–24]. However, there were no studies relating these events with the activation of the three pathways of ERS. Our results suggest that HgCl_2_KI partially activated the PERK pathway during the first 48 h through the activation of two pathways.

One pathway involved PERK-immediate substrate nuclear factor erythroid 2-related factor 2 (Nrf2). Nrf2 is independent of eIF2α phosphorylation, and it is one of the primary regulators of cytoprotective responses to oxidative stress, as we can observe in the result above [25–27]. Thus, Nrf2 could act as a potent activator for the transcription of antioxidant enzymes like glutathione S-transferase (GST) and GR as we can observe in the temporary result of this study, particularly 48 h after mercury intoxication [28,29]. These mechanisms have a relationship with mercury bioaccumulation process in kidney cells more than antioxidants or detoxifying compensatory action. Moreover, it has an association between GADD153 and oxidative stress process because it induces the overexpression and over-activity of endoplasmic reticulum oxidoreductin 1 (ERO-1) which enhances H_2_O_2_ production and cytoplasm Ca^2+^ overload to promotes oxidative stress [30,31]. Also, it has been reported that Sirt1/Nrf2/OH-1 pathway is insufficient to respond to the mercury chloride causes oxidative stress because HgCl_2_ blocks positive feedback loop Sirt1/Keap1/Nrf2/ARE that is an antioxidant pathway [8].

The second mechanism involved the phosphorylating eIF2α which attenuates global protein translation and also it activates ATF-4-dependent transcription. We proposed that the second route is progressively activating from 48 h until 72 h because there is a rise in the expression of ATF-4, which can induce the expression of GADD-34, a protein that is associated with protein phosphatase 1 (PP1) and promotes dephosphorylation of eIF2α [32]. Thus, it attenuates the PERK/eIF2α pathway to promote cell death. After 72 h of mercury intoxication, ATF-4 and GADD-153 have the same pattern of expression. Thus ATF-4 mediated GADD-153 translation through GADD 34 [33]. Meanwhile, GADD34 is a target molecule of ATF6 and IRE1α pathways. In our result, IRE1α had the same pattern expression of GADD34, but ATF6 presented a stepwise expression increase, and so did XBP-1. These molecular mechanisms promote the synthesis of new ERS mediators and cell-death promoters like GADD-153 [34,35]. 72 h after mercury exposition, the apoptosis process is activated because the IRE1α kinase domain forms a complex with adaptor protein TNF receptor-associated factor 2 (TRAF2), the apoptosis signal regulator kinase 1 (ASK1), and the c-Jun kinases (JNK) [36]. This hypothesis is supported because mercury reduces the expression of BCL_2_ y BCL_XL_ with an increase of BAX, BAK, and caspase-12 [8,16,37]. This molecular event participates in the formation of the mitochondrial transition pore (MTP), which allows the release of cytochrome c from mitochondria to the cytoplasm to form the apoptosome [10]. This idea is supported because our results show the overexpression patter of GAD153 agrees with the expression of caspase 12 and the over-activity of caspase 3.

Finally, the increase of the oxidative and ERS promotes the over-expression of annexin-V with evident histological damage characterized by in the hematoxylin–eosin stain was edema, the cellular atrophy of distal and proximal tubules, distortion of cellular continuity, nucleus loss, and hyperchromatic nuclei. All of these biochemical, molecular, and morphological change promotes renal dysfunction.

## 5. Conclusion

We conclude that ERS participates in HgCl_2_ caused AKI because the PERK/eIF2α branch is activated during the first 48 has a protective but not effective response. Also, the activation of the PERK/ATF-4 branch allows that ATF-4, ATF-6, and IRE1α pathways activate a cell-death promoter response through GADD-153 and the subsequent activation of caspases 12 and 3, which mediate the death and renal dysfunction.
